# Comment: Characterization of Two Historic Smallpox Specimens from a Czech Museum

**DOI:** 10.3390/v9100276

**Published:** 2017-09-28

**Authors:** Ashleigh F. Porter, Ana T. Duggan, Hendrik N. Poinar, Edward C. Holmes

**Affiliations:** 1 Marie Bashir Institute for Infectious Diseases and Biosecurity, Charles Perkins Centre, School of Life and Environmental Sciences and Sydney Medical School, The University of Sydney, Sydney, NSW 2006, Australia; ashleigh.porter@sydney.edu.au; 2 McMaster Ancient DNA Centre, Department of Anthropology, McMaster University, Hamilton, ON L8S 4L9, Canada; duggana@mcmaster.ca (A.T.D.); poinarh@mcmaster.ca (H.N.P.); 3 Michael G. DeGroote Institute for Infectious Disease Research and the Department of Biochemistry, McMaster University, Hamilton, ON L8S 4L8, Canada

**Keywords:** smallpox, variola virus, evolution, ancient DNA, molecular clock, phylogeny

## Abstract

The complete genome sequences of two strains of variola virus (VARV) sampled from human smallpox specimens present in the Czech National Museum, Prague, were recently determined, with one of the sequences estimated to date to the mid-19th century. Using molecular clock methods, the authors of this study go on to infer that the currently available strains of VARV share an older common ancestor, at around 1350 AD, than some recent estimates based on other archival human samples. Herein, we show that the two Czech strains exhibit anomalous branch lengths given their proposed age, and by assuming a constant rate of evolutionary change across the rest of the VARV phylogeny estimate that their true age in fact lies between 1918 and 1937. We therefore suggest that the age of the common ancestor of currently available VARV genomes most likely dates to late 16th and early 17th centuries and not ~1350 AD.

## 1. Introduction

Pajer et al. [1] recently characterized two human smallpox specimens from the Czech National Museum in Prague, retrieving the complete genomes of the causative variola virus (VARV) in both cases. The first specimen, V1588, consisted of a 10 cm^2^ piece of skin with pock lesions, while the second, V563, comprised an intact forearm and foot from a child displaying the distinctive smallpox rash. Although no documentation nor history was available for either specimen, their age was inferred by the degree of d-, l-aspartic acid racemization to be 1809–1889 (mean 1850) for V1588 and 1939–1969 (mean 1942) for V563 [1]. With these sequences, the authors of this study then estimated the rate and time-scale of VARV evolution, suggesting that the available VARV strains share a common ancestor that existed around 1350 AD. This is older than the time to common ancestry (1588–1645) previously determined by Duggan et al. [2] following the description of a complete VARV genome (VD21) from a 17th century Lithuanian mummy, and implies that smallpox has greater antiquity in Europe. Herein we query the estimated ages of V1588 and V563 and hence the time-scale of smallpox evolution presented by Pajer et al. [1], particularly as more recent studies have also utilized V1588 and V563 to date the antiquity of VARV [3].

## 2. Results and Discussion

The racemization of amino acids used by Pajer et al. [1] depends on many factors including the pH (both strong acidity and alkali), temperature, and concentration of various solutes in solution [4]. For example, amino acids will undergo racemization in the presence of heavy metals, such as copper, nickel and lead [5]. Given this, and without key information about the pH of the fixatives used, it is interesting to note that sample V1588, which has a 2.5× higher d/l Asp ratio than V563 has also 2× the amount of copper, 10× the amount of nickel and 5× the amount of lead. Control samples used to calibrate the d/l ‘clock’ show considerable variance; for example, in samples known to be between 119 and 122 years old the d/l ratio ranged from 0.086 to 0.158 (0.122 +/− 0.072). Hence, we believe that it is unwise to assign an age to either of these samples without proper archival information.

**Figure 1:**
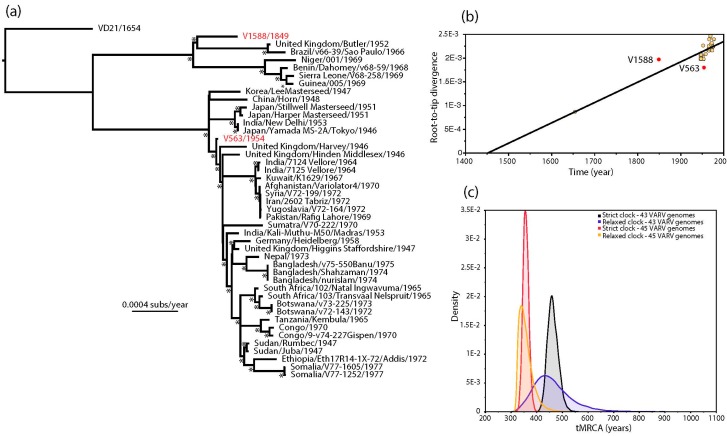
(**a**) Maximum likelihood (ML) phylogenetic tree of 45 complete genomes of VARV. The phylogeny was based on a complete genome alignment of 181,261 bp constructed using a combination of MAFFT [11] and GBlocks [12] pruning to remove ambiguously aligned regions. The phylogeny was inferred using PhyML [13] assuming the General Time Reversible (GTR) model of nucleotide substitution, with a proportion of invariable sites (I) and a gamma distribution (Г) of among-site rate variation. All horizontal branch lengths are scaled according to the number of nucleotide substitutions per site, and bootstrap values >90% are marked with a * symbol. The tree is rooted using a sequence obtained from a Lithuanian mummy (VD21) and V1588 and V563 are shown in red; (**b**) regression of root-to-tip genetic distances from the ML tree against their sampling year. V1588 and V563 are shown in red. This analysis was performed using the TempEst program [14]; (**c**) bayesian estimates of times to common ancestry in VARV. The ’45 VARV’ estimates utilized the tip times in the full 45 genome sequence data set, incorporating mean age estimates of 1849 and 1954 for V1588 and V563, respectively, that are taken from the distribution of possible racemization-based dates provided by Pajer et al. [1]. The ’43 VARV’ estimates are those in which a uniform prior distribution with bounds of 0 and 1.0E10 was specified for the ages of V1588 and V563, with these and all other parameter values inferred from the remaining 43 sequences in the alignment. All these analyses were performed using the BEAST package [8], run for 100 million generations, and employing a constant population size (XML files are available in the supplementary material).

The estimated ages of V1588 and V563 provided by Pajer et al. [1] also conflict with the strongly clock-like evolution of VARV [2,6,7]. This discrepancy is apparent in a (non-clock) maximum likelihood (ML) tree of 45 complete VARV genomes in which V563 and V1588 occupy anomalous positions (Figure 1a). In particular, V1588 seemingly falls closer to the tips of the tree (i.e., the present) than V563 even though it was supposedly sampled approximately 100 years earlier. This impression is confirmed by a regression of root-to-tip genetic distances on the ML tree against sampling year, in which V1588 appears to be evolving anomalously rapidly and V563 anomalously slowly (Figure 1b). Although it is theoretically possible that the clock-like evolution of VARV breaks down in V1588 and V563, it is striking that both these viruses came from the same study and their ages were estimated in a similar manner.

Given the strongly clock-like evolution present in the remainder of the VARV phylogeny we employed a Bayesian Markov chain Monte Carlo method [8] to estimate the ages of V1588 and V563. First, we repeated the molecular clock dating analysis of Pajer et al. [1], using ages of 1849 and 1954 as the tip dates for V1588 and V563, respectively, as these represent the means of the distributions of possible racemization-estimated dates provided by these authors [1]. Under both strict and relaxed (uncorrelated lognormal) molecular clocks this resulted in lower rates of evolutionary change (means of 5.44 and 5.89 × 10^−6^ nucleotide substitutions per site, per year, respectively) and slightly older mean times to the most recent common ancestor (tMRCA; means of 1514 and 1515, respectively) than previously obtained by Duggan et al. [2], although more recent than those obtained by Pajer et al. [1] (Figure 1c) (Table 1). Next, we estimated the ages of V1588 and V563 by specifying a prior distribution for the age of both viruses using the evolutionary rate and date information from the 43 remaining VARV genomes. As expected, both strict and relaxed molecular clocks gave evolutionary rates and divergence times very similar to those obtained by Duggan et al. [2]—at 8.27 and 8.73 × 10^−6^ nucleotide substitutions per site, per year, respectively (Table 1). More importantly, the ages of V1588 and V563 were estimated to be 1921 and 1918, respectively, under a strict molecular clock and 1937 and 1933 under a relaxed clock. Hence, if we assume that VARV evolves in a strongly clock-like manner then we can safely infer that both V1588 and V563 likely date to similar times in the 20th century, with no compelling evidence that V1588 is 160 years old. The use of incorrectly dated sequences has previously been shown to adversely impact studies of virus evolution [9,10], and hence should be considered in all exercises in molecular clock dating.

In sum, we suggest that the rates of nucleotide substitution and time-scale of VARV proposed by Duggan et al. [2] are still the best evolutionary description of this historically important human pathogen, with no compelling evidence that available strains of VARV share a common ancestor as early as ~1350 AD. Given the highly variable branch lengths between VARV and other mammalian poxviruses, which likely result from very different rates of evolutionary change, we also believe it is unwise to use molecular clock methods to date the divergence between VARV and its closest animal relatives [3].

## Figures and Tables

**Table 1:** Results of the Bayesian analysis of the evolutionary history of variola virus (VARV).

Data Set and Model	Substitution Rate (×10^−6^ subs/site/year)	tMRCA VARV
^1^ Strict clock—45 genomes	5.44 (4.73–6.16)	1514 (1554–1645)
Relaxed clock—45 genomes	5.89 (4.19–7.67)	1515 (1366–1642)
^2^ Strict clock—43 genomes	8.27 (7.48–9.10)	1620 (1598–1642)
Relaxed clock—43 genomes	8.73 (7.02–10.02)	1619 (1566–1654)

^1^ Based on the 45 VARV genome data used by Pajer et al. [1] including V1588 and V563. ^2^ Based on 43 VARV genomes excluding V1588 and V563 for which a prior distribution is given on their age. Strict = strict molecular clock; Relaxed = relaxed (uncorrelated lognormal) molecular clock.

## Supplementary Materials

The following are available online at www.mdpi.com/1999-4915/9/10/276/s1, XML files associated with the BEAST analysis of the ’43 VARV’ and ’45 VARV’ genome data sets.
